# Posthumous Volume of Dr. Drake’s Treatise on the Diseases, Etc., of the Interior Valley

**Published:** 1853-04

**Authors:** 


*Posthumous volume of Dr. Drake's treatise on the diseases, etc., of the interior valley.* — We have alluded, in another article, to an announcement by the relatives of the late Dr. Drake, that the materials for the second vol. of the work on the diseases, etc., of the interior valley, were left by the author in a state to admit of its publication. Since that article was written, we have been informed that the labor of preparing the volume has been entrusted to S. Hanbury Smith, M. D., late professor of the principles and practice of medicine in the Starling Medical College, and more recently superintendent of the Ohio State Lunatic Asylum. Dr. Smith will bring to this duty a willing heart, and the ability as well as disposition to do justice to the unfinished undertaking of his deceased friend.

